# Indole-3-acetic acid and chenodeoxycholic acid attenuate TLR4/NF-κB signaling and endoplasmic reticulum stress in valproic acid-induced neurotoxicity

**DOI:** 10.3389/fphar.2025.1570125

**Published:** 2025-03-24

**Authors:** Wedad S. Sarawi, Ahlam M. Alhusaini, Ghada S. Barwaished, Myasah M. Altamimi, Iman H. Hasan, Amjad S. Aljarboa, Norah K. Algarzae, Saleh A. Bakheet, Samiah A. Alhabardi, Sheikh F. Ahmad

**Affiliations:** ^1^ Department of Pharmacology and Toxicology, College of Pharmacy, King Saud University, Riyadh, Saudi Arabia; ^2^ College of Pharmacy, King Saud University, Riyadh, Saudi Arabia; ^3^ Department of Physiology, College of Medicine, King Saud University, Riyadh, Saudi Arabia; ^4^ Department of Pharmaceutics, College of Pharmacy, King Saud University, Riyadh, Saudi Arabia

**Keywords:** valproic acid neurotoxicity, indole-3-acetic acid, CDCA, GRP78, CHOP, NF-kappa B

## Abstract

Valproic acid (VA) is a commonly prescribed medication for epilepsy and other neurological conditions. Although effective, VA use can lead to neurotoxicity, especially with chronic use. This study aimed to investigate the potential neuroprotective properties of indole-3-acetic acid (IAA) and chenodeoxycholic acid (CDCA) in an animal model of VA-induced brain injury. Rats received intraperitoneal injections of VA at a dose of 500 mg/kg/day for 3 weeks. Concurrently, they were orally treated with IAA (40 mg/kg/day) and/or CDCA (90 mg/kg/day). The results showed significantly increased oxidative stress and inflammation markers in the VA-exposed group indicated by the reduced levels of glutathione (GSH, P < 0.0001) and superoxide dismutase (SOD, P < 0.01) and the elevated inflammatory cytokines Interleukin-6 (IL-6, P < 0.0001) and tumor necrosis factor-alpha (TNFα, P < 0.01). VA also induced nuclear factor kappa B (NF-κB, P < 0.01), toll-like receptor 4 (TLR4, P < 0.05), and endoplasmic reticulum (ER) stress markers, as evidenced by increased immunoreactivity of GRP78 (glucose-regulated protein 78, P < 0.0001), transcription factor 6 (ATF-6, P < 0.05) and CHOP (C/EBP homologous protein, P < 0.0001). Treatment with IAA or CDCA attenuated VA-induced neurotoxicity, to a variable extent, by improving oxidative, inflammatory, and ER stress markers. This study demonstrates that IAA and CDCA exert protective effects against VA-induced neurotoxicity by mitigating oxidative stress, inflammation, and ER stress. Further investigations are recommended to validate these findings in other neurotoxicity models.

## 1 Introduction

Natural medicines have gained significant attention in the treatment of drug-induced neurotoxicity and neurodegeneration due to their potential protective effects and fewer side effects compared to conventional treatments (Sarkar et al., 2024). While herbal treatments show promise, conventional medications like valproic acid (VA) remain crucial in managing neurological disorders such as epilepsy, bipolar disorder, migraine, and other neuropsychiatric disorders VA is a commonly used medication to control (Ghodke-Puranik et al., 2013; Patel and Nagalli, 2024). It exerts its action by inhibiting succinic semialdehyde dehydrogenase, leading to increased γ-aminobutyric acid (GABA) levels and enhanced GABAergic neurotransmission. It also reduces glutamatergic transmission and inhibits voltage-gated sodium channels, contributing to brain cortical inhibition (Romoli et al., 2019). VA is generally considered safe but has a narrow therapeutic index (Methaneethorn, 2018). While VA exhibits a neuroprotective effect at therapeutic doses and duration in traumatic brain injury (Wakam et al., 2021), and spinal cord injury (Lee et al., 2014), exceeding these doses and duration can negate these benefits.

Chronic exposure to VA can cause toxicities, including hyperammonemia, encephalopathy, CNS depression, and hepatotoxicity (Sztajnkrycer, 2002). Serum valproate concentrations of 180 mg/L or greater are often associated with CNS dysfunction, characterized by tremors, agitation, brain edema, and neuronal damage (Liu et al., 2024). An overexposure to VA may cause VA-induced brain injury and neuronal toxicity through several mechanisms, including oxidative stress and inflammation (Chaudhary and Parvez, 2012; Taleb et al., 2021). VA caused elevation of protein carbonyl and lipid peroxidation (LPO), well-known biomarkers for oxidative stress, along with a reduction of the non-enzymatic antioxidants including glutathione (GSH) and n-protein thiol activity (NP-SH). Other antioxidant enzymes like superoxide dismutase (SOD) and catalase (CAT) also showed a significant reduction in their activities (Chaudhary and Parvez, 2012).

More recent studies have highlighted the potential neuroprotective effects of certain compounds, indole-3-acetic acid (IAA) and chenodeoxycholic acid (CDCA) emerging as promising drug candidates (Bazzari et al., 2019; Ji et al., 2020). IAA is one of the key auxin plant hormones generated from the metabolism of the essential amino acid tryptophan which is considered a precursor for various neurotransmitters such as serotonin (Ferrari et al., 2014). CDCA is a primary bile acid synthesized from cholesterol in the liver. It has emerged as a significant compound with potential neuroprotective and anti-inflammatory properties, underscored by its ability to interact with the farnesoid X receptor (FXR) (Bazzari et al., 2019). The neuroprotective potential of IAA and CDCA has not been thoroughly studied in the context of VA-induced neurotoxicity. Thus, this research seeks to elucidate the mechanisms through which VA induces neurotoxicity and evaluate the neuroprotective properties of IAA and CDCA in the valproic acid-induced neuronal toxicity rat model.

## 2 Methods

### 2.1 Animals

The animal care center at King Saud University’s College of Pharmacy provided thirty male Wistar albino rats (mean weight 155 ± 25 g). Animals were kept in conventional polypropylene cages under controlled environmental conditions and given *ad libitum* access to normal rodent food and water. Before commencing any experimental procedures, a 1-week acclimatization phase was allowed. All experimental methods were carried out strictly according to the Institutional Animal Care and Use Committee (IACUC) and approved by the Committee of Research Ethics at KSU (Approval No.: KSU-SE-20–72).

### 2.2 Experimental design

**FIGURE 1:**
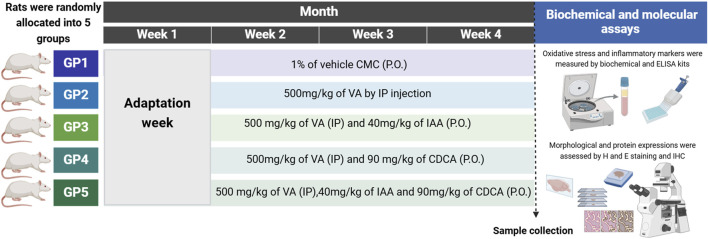
Schematic representation for the experimental design, timeline, and subsequent workflow. The dashed line represents the time of sample collection. GP: group, CMC: carboxymethylcellulose, P.O.: orally, VA: valproic acid, IP: intraperitoneal, indole-3-acetic acid (IAA) and chenodeoxycholic acid (CDCA), H and E: hematoxylin and eosin. ELISA: enzyme-linked immunosorbent assay. IHC: immunohistochemistry. Created by BioRender.

Rats were randomly divided into five groups with six rats in each, group 1 (control) was given an equivalent volume of 1% carboxymethylcellulose (CMC) orally for 3 weeks. Groups 2 to 5 were injected daily with an intraperitoneal dose of VA at 500 mg/kg for 21 days (Ezhilarasan and Mani, 2022; Terim Kapakin et al., 2024). Groups 3 to 5 were orally treated with 40 mg/kg/day of IAA (Oliveira et al., 2007), 90 mg/kg/day of CDCA (Bazzari et al., 2019), or a combination of both IAA and CDCA an hour post-VA dose for 21 days as shown in Figure 1.

At the end of treatment, rats were weighed and humanely euthanized by a rising level of carbon dioxide, followed by decapitation. Whole trunk blood was taken and spun at 3,000 rpm for 30 min at 4°C to obtain sera. Brain tissue was carefully resected from the skull, cortex was localized and rinsed with ice-cold phosphate-buffered saline (PBS) then divided into two parts; one was preserved in 10% formaldehyde for histopathological and immunohistochemical (IHC) investigations. The second part was homogenized in PBS (5% w/v) with a tissue homogenizer, centrifuged at 3,000 rpm for 30 min at 4°C, and the supernatants were collected and frozen at −80°C for subsequent biochemical and molecular analysis.

### 2.3 Assessment of oxidative stress markers in the brain tissue

Colorimetric assays were used to determine the level of reduced GSH (Cat no. EEA020, Invitrogen) and SOD activity (Cat no. EIASODC, Invitrogen). The optical density was determined by a spectrophotometer at 405 nm for GSH and 450 nm for SOD as instructed by the obtained kits.

### 2.4 Assessment of inflammatory stress markers in the brain tissue

A sandwich enzyme-linked immunosorbent assay (ELISA) was conducted to measure the levels of IL-6 and TNFα in the cortical brain tissue homogenates. The ELISA kit for IL-6 was obtained from Sigma-Aldrich (Cat no. RAB0311), while the TNFα kit was purchased from AFG Bioscience (Cat no. EK720127). The absorbance was measured at 450 nm using a microplate reader for both assays.

### 2.5 Brain tissue histopathological evaluation

Brain tissue was prepared for paraffin embedding and subsequently sectioned at a thickness of 5 µm. These sections were dewaxed in xylene and rehydrated through a series of graded ethanols (100%, 95%, and 70%) and distilled water, each for 10 min. Following rehydration, some sections were stained with hematoxylin, rinsed, counterstained with eosin (H&E), dehydrated, and mounted for examination.

### 2.6 Assessment of TLR4/NF-κB and ER stress markers in the brain tissue

On the first day, slides were dewaxed in xylene and rehydrated as mentioned in the histopathological evaluation. Antigen retrieval was performed by immersing slides in 10 mM citric acid buffer (pH 6) and boiling. Endogenous peroxidase activity was blocked using 0.3% hydrogen peroxide. Subsequently, sections were permeabilized, blocked, and incubated overnight with primary antibodies against NF-κB p65 (ab16502, Abcam), TLR4 (bs-20594R, Bioss), GRP78 (K200061M, SolarBio), ATF6 (K001590P, SolarBio) and CHOP (K107245P, SolarBio). The following day, sections were washed and incubated with biotinylated secondary antibodies. An Avidin-Biotin Complex (ABC, Vector, PK-4000) was then added to amplify the immunoreactive signal. Diaminobenzidine (DAB) (Vector, SK-4100) was used as chromogen for visualization. Finally, all sections were counterstained, mounted, and examined under a brightfield microscope.

### 2.7 Statistical analysis

Data were expressed as mean ± SEM. Differences between groups were analyzed using Prism 9. One-way ANOVA followed by Tukey’s *post hoc* test was used to compare the means between the five experimental groups. The percentage of degenerative neurons (%) in different treatment groups and the staining intensity were calculated using ImageJ software. A P-value less than 0.05 was considered statistically significant.

## 3 Results

### 3.1 Effect of IAA and CDCA on the histological changes observed in VA-induced neurotoxicity

**FIGURE 2:**
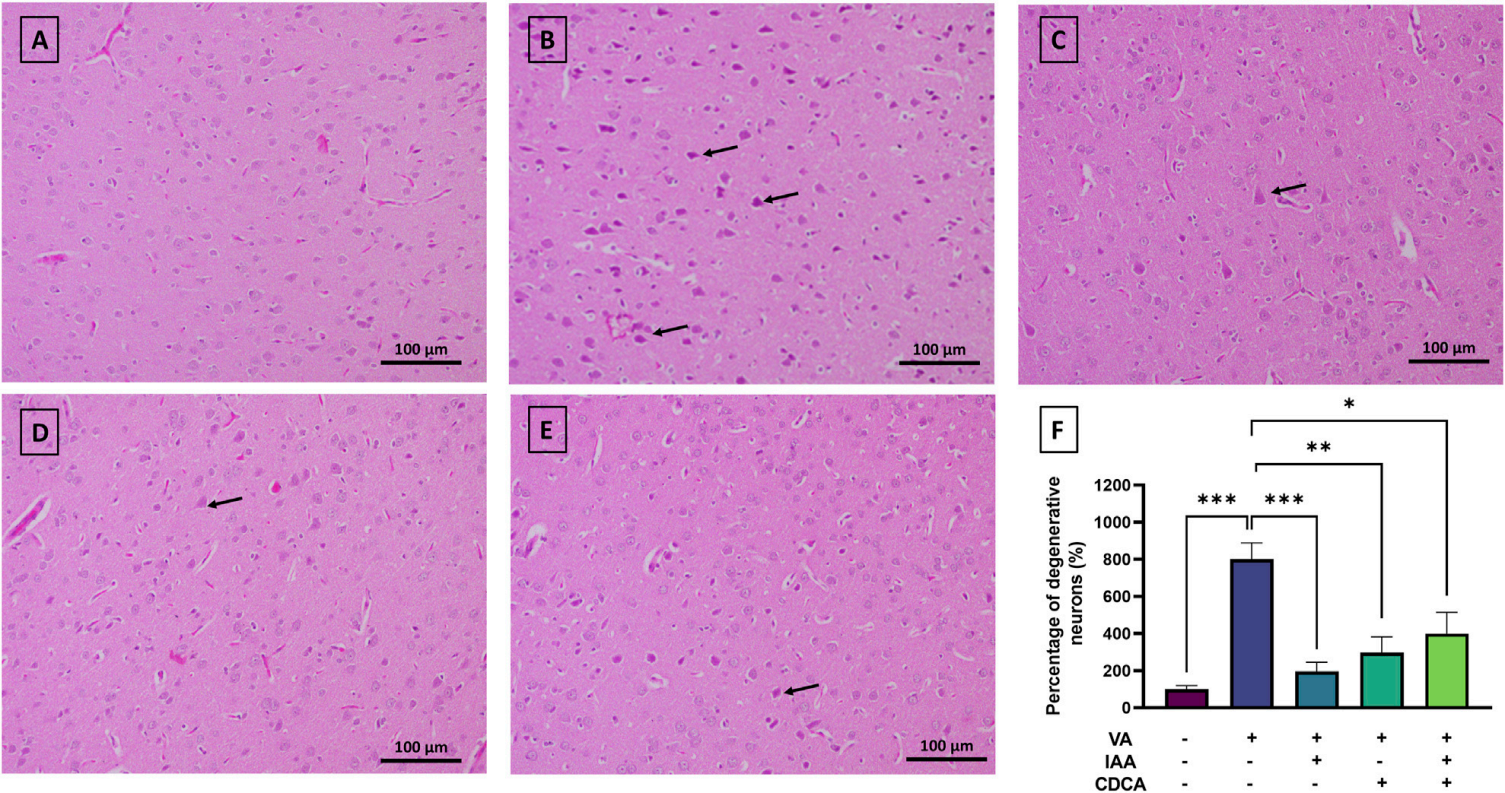
Representative histopathological changes of rat cortex in VA-induced neurotoxicity. Scale bar: 100 μm, magnification ×200. **(A)** Brain cortex of normal control rats displaying normally H&E-stained neurons. **(B)** Brain cortex of rats intoxicated with VA exhibiting numerous degenerated neurons, indicated by pyknosis (arrows), interspersed among a few scattered normal neurons **(C–E)** Brain cortex of VA-intoxicated rats treated with **(C)** IAA, **(D)** CDCA, or **(E)** concurrent IAA + CDCA demonstrating a reduction in VA-induced neuronal degeneration, as evidenced by a lower number of degenerated neurons (arrows) and a greater number of normal neurons. **(F)** Percentages of degenerative neurons (%) in different treatment groups are calculated by ImageJ. Values are expressed as mean ± SEM. ***P < 0.001, **P < 0.01, *P < 0.05, (*n* = 4). H and E: hematoxylin and eosin, VA: valproic acid, IAA: indole-3-acetic acid, and CDCA: chenodeoxycholic acid.

Rats were exposed to VA, with and without IAA and CDCA treatments, for three consecutive weeks. Histological staining was used to assess the pathological changes induced by VA exposure and the effects of IAA and CDCA treatments as shown in Figure 2. The control group exhibited normal nerve cell morphology in the cortical brain region. VA exposure significantly increased the number of degenerated (pyknotic) neurons (***P < 0.001). Treatment with IAA and CDCA individually significantly reduced neuronal degeneration compared to the VA group (***P < 0.001 and **P < 0.01, respectively), demonstrating protective effects against VA-induced damage. While rats treated concurrently with IAA and CDCA also showed a significant reduction in degenerated neurons compared to VA-exposed rats (*P < 0.05), this protective effect was less pronounced than that of the individual IAA or CDCA treatments.

### 3.2 Effect of IAA and CDCA on the oxidative stress in VA-induced neurotoxicity

**FIGURE 3:**
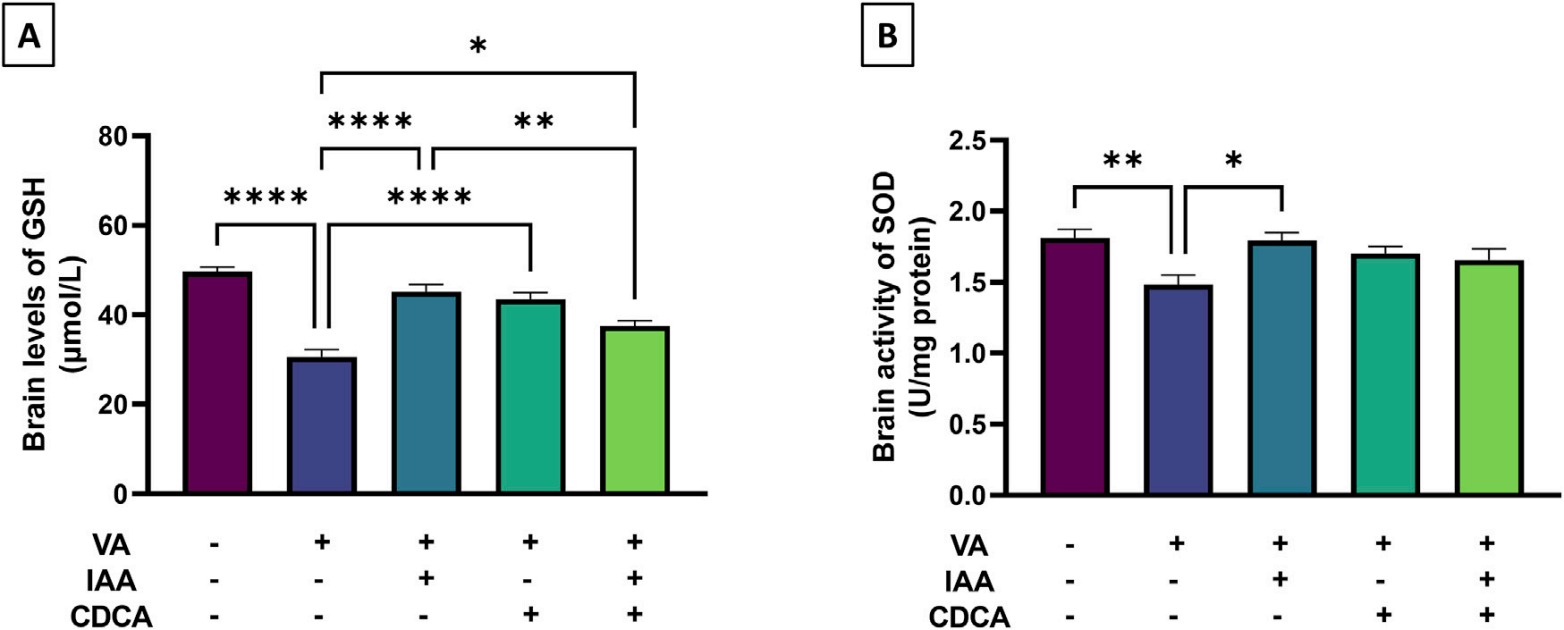
Effects of IAA and CDCA on the cortical level of **(A)** reduced glutathione (GSH) and activity of **(B)** superoxide dismutase (SOD) in VA-induced neurotoxicity in rats. Values are expressed as mean ± SEM. ****P < 0.0001, **P < 0.01, *P < 0.05, (n = 6). VA: valproic acid, IAA: indole-3-acetic acid, and CDCA: chenodeoxycholic acid.

Antioxidants like GSH and SOD were assessed during VA exposure and after treatments. As shown in Figure 3, cortical level of GSH decreased significantly with VA treatment, (***P < 0.001) compared to the control, indicating increased oxidative stress. Notably, the use of IAA, CDCA and their combination besides VA significantly reverse the VA effect by increasing GSH level (****P < 0.0001, ****P < 0.0001, *P < 0.05, respectively). However, the combined treatment of IAA and CDCA showed a less pronounced effect when compared to the IAA-treated group regarding increasing GSH level (**P < 0.01). Additionally, the SOD activity was significantly reduced by VA treatment (**P < 0.01), and upon treatment with IAA alone, the reduction was reversed considerably (*P < 0.05).

### 3.3 Effect of IAA and CDCA on inflammatory cytokines in VA-induced neurotoxicity

**FIGURE 4:**
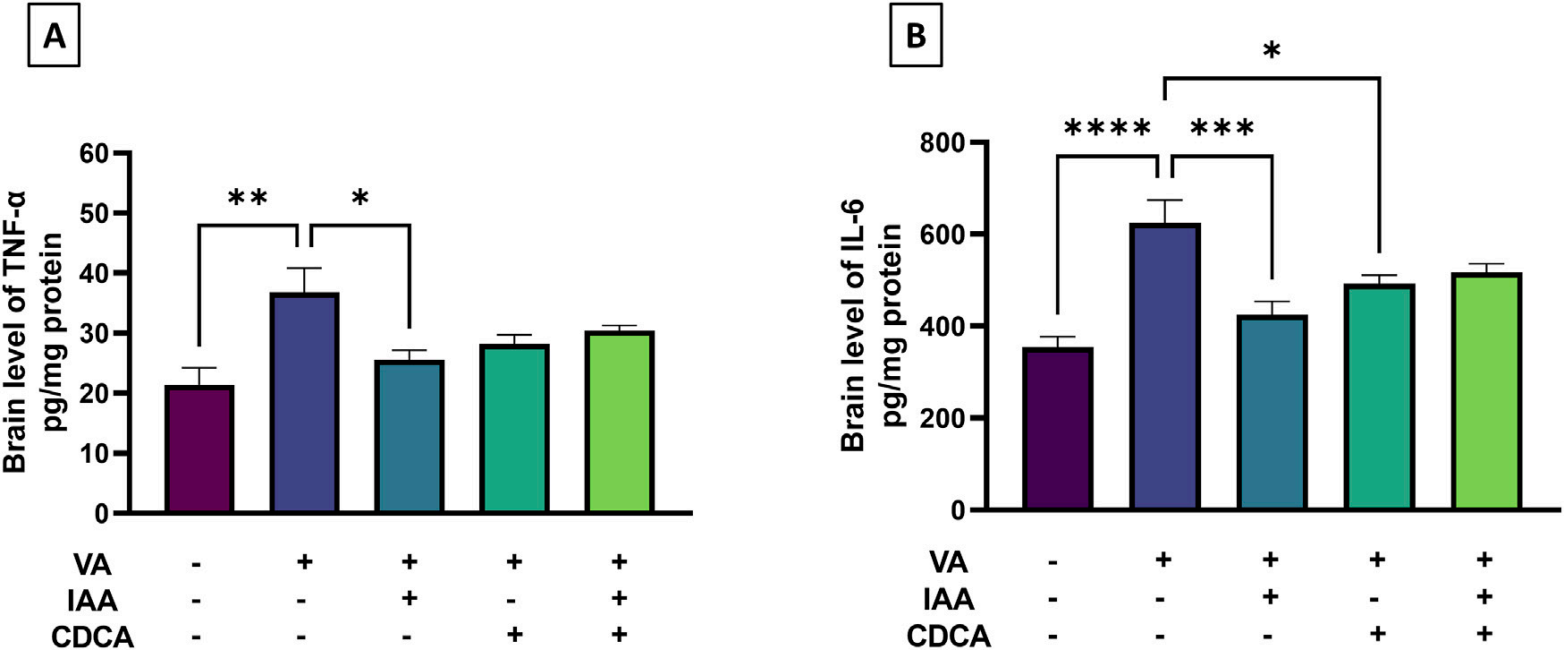
Effects of IAA and CDCA on the cortical levels of **(A)** tumor necrosis factor-α (TNF-α) and **(B)** interleukin-6 (IL-6) in VA-induced neurotoxicity in rats. Values are expressed as mean ± SEM. ****P < 0.0001, ***P < 0.001, **P < 0.01, *P < 0.05, (*n* = 6). VA: valproic acid, IAA: indole-3-acetic acid, and CDCA: chenodeoxycholic acid.

To assess VA-induced brain inflammation, brain cortical levels of TNF-α and IL-6 were measured as markers. Figure 4 showed a marked increase in TNF-α in the VA-treated group compared to controls (**P < 0.01). This increase was significantly attenuated by IAA treatment (*P < 0.05). CDCA, alone or in combination with IAA, also reduced TNF-α levels, but not significantly. Moreover, IL-6 levels were markedly increased in the VA-exposed group compared to controls (****P < 0.0001). Both individual treatments of IAA and CDCA decreased cortical brain levels of IL-6 compared to VA alone (***P < 0.001 and *P < 0.05, respectively), but this was not the case with combined treatment.

### 3.4 Effect of IAA and CDCA on cortical expression of NF-κB, TLR4, and ER stress markers in VA-induced neurotoxicity

#### 3.4.1 NF-κB expression

**FIGURE 5:**
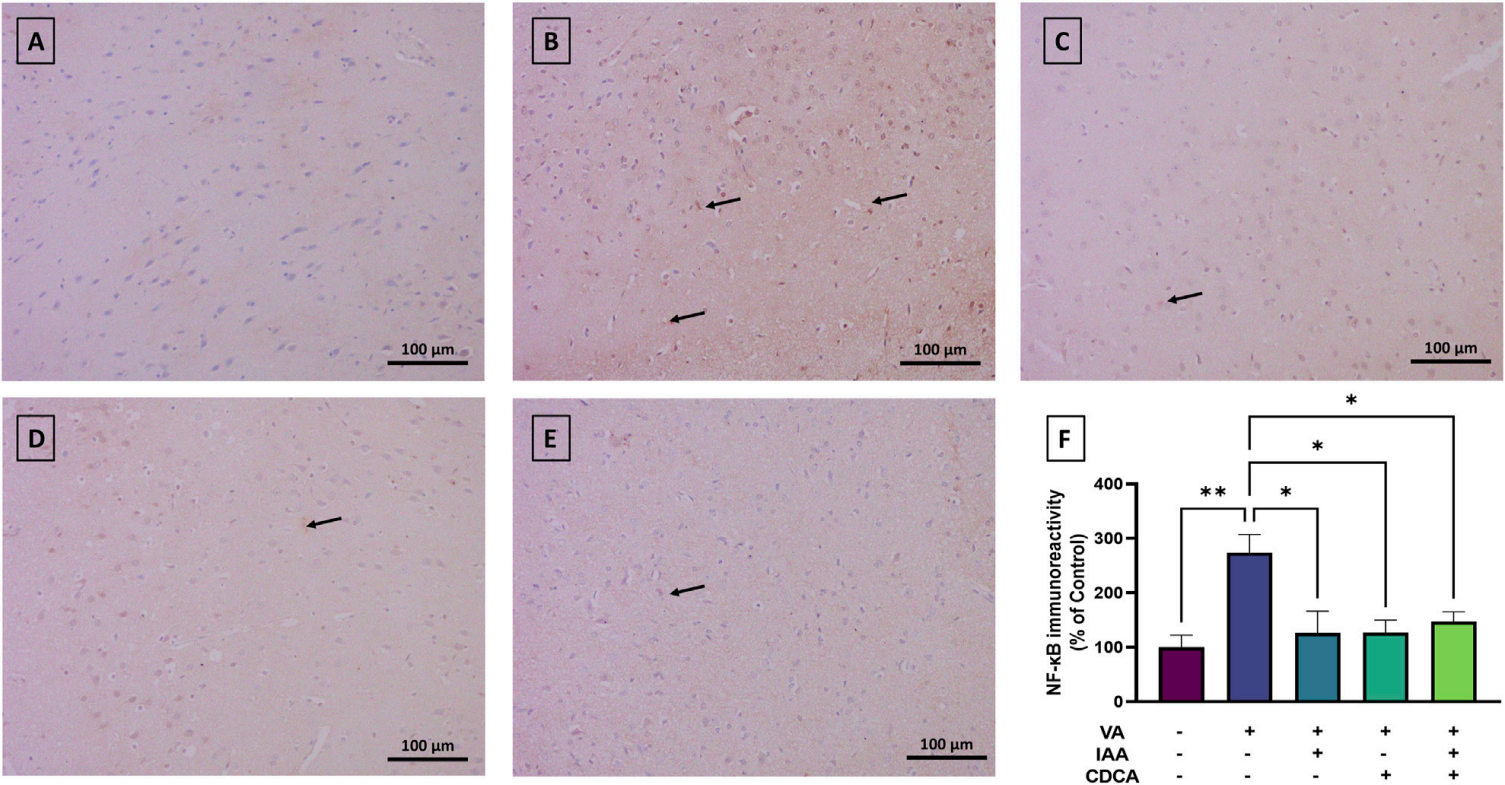
Immunoreactivity of NF-κB in brain cortex during VA neurotoxicity and after IAA and CDCA treatments. Scale bar: 100 μm, magnification ×200. **(A)** Brain cortex of normal control rats showing normal immune reactive signal for NF-κB. **(B)** Brain cortex of rats intoxicated with VA exhibiting a moderate upregulation in immunoreactivity (arrows). **(C–E)** Brain cortex of VA-intoxicated rats concurrently treated with **(C)** IAA, **(D)** CDCA, and **(E)** IAA + CDCA demonstrating significant reductions in VA-induced upregulation in NF-κB expression. **(F)** Quantification of NF-κB expression across treatment groups using ImageJ. Values are expressed as mean ± SEM. **P < 0.01, *P < 0.05, (*n* = 4). NF-κB: nuclear factor kappa B, VA: valproic acid, IAA: indole-3-acetic acid, and CDCA: chenodeoxycholic acid.

NF-κB immunostaining revealed low baseline expression in the cortical brain sections of control rats (Figure 5). However, NF-κB immunoreactivity significantly increased with VA exposure compared to controls (**P < 0.01). Treatment with IAA or CDCA in conjunction with VA markedly reduced the immunoreactive signal (*P < 0.05). Similarly, combination treatments also reduced NF-κB immunoreactivity relative to VA alone (*P < 0.05).

#### 3.4.2 TLR4 expression

**FIGURE 6:**
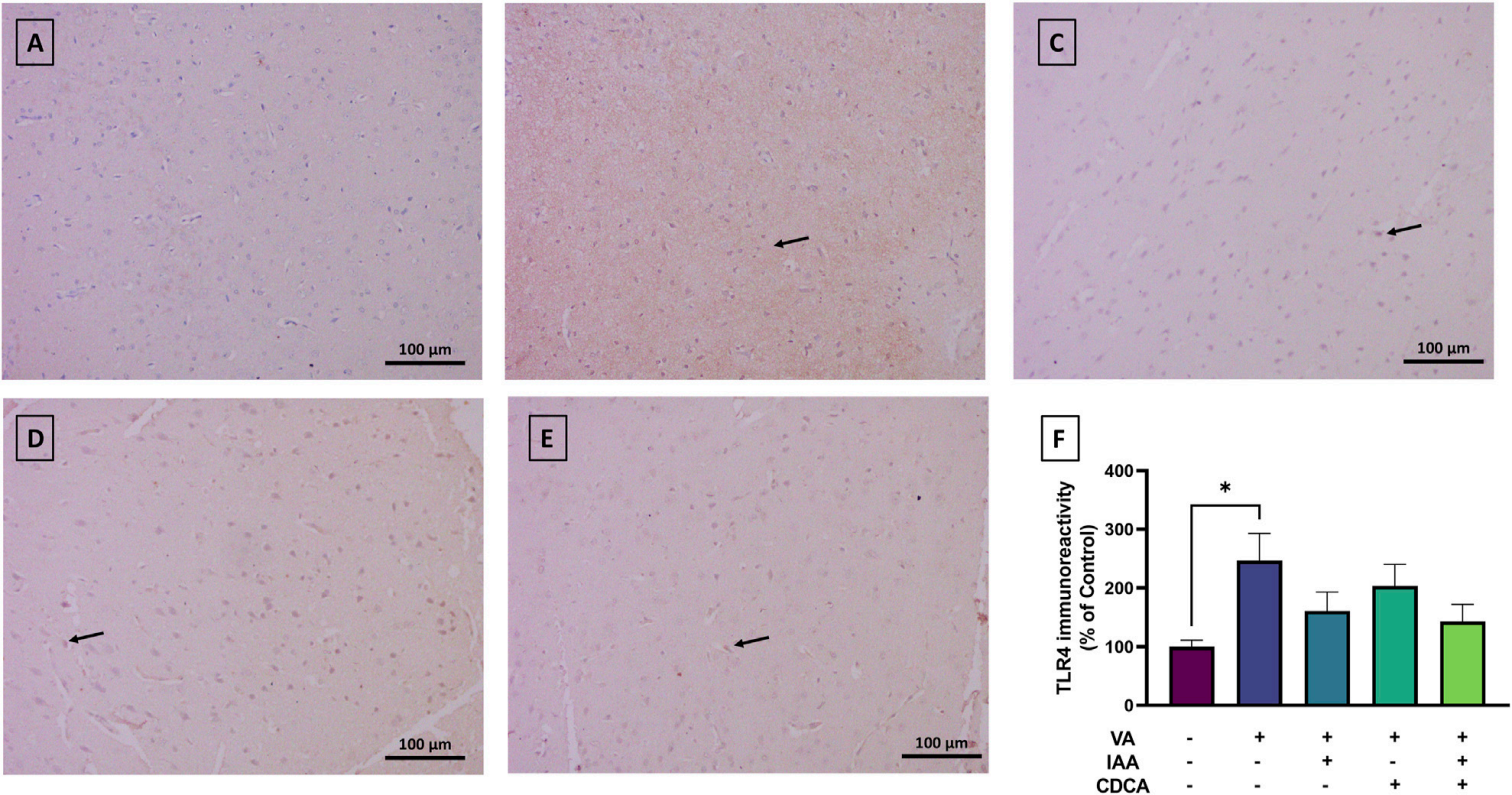
Immunoreactivity of TLR4 in brain cortex during VA neurotoxicity and after IAA and CDCA treatments. Scale bar: 100 μm, magnification ×200. **(A)** Brain cortex of normal control rats showing normal immune reactive signal for TLR4. **(B)** Brain cortex of rats intoxicated with VA exhibiting a mild upregulation in immunoreactivity (arrows). **(C–E)** Brain cortex of VA-intoxicated rats concurrently treated with **(C)** IAA, **(D)** CDCA, and **(E)** IAA + CDCA demonstrating some reductions in VA-induced upregulation in TLR4 expression. **(F)** Quantification of TLR4 expression in different treatment groups using ImageJ. Values are expressed as mean ± SEM. *P < 0.05, (*n* = 4). TLR4: toll-like receptor 4, VA: valproic acid, IAA: indole-3-acetic acid, and CDCA: chenodeoxycholic acid.

Cortical brain sections from the normal control group revealed low TLR4 immunoreactivity (Figure 6). However, the VA administration revealed a marked increase in TLR4 expression (*P < 0.05). Treatment with IAA, CDCA, or their combination in conjunction with VA reduced the immunoreactivity but the results were not significant (P > 0.05).

#### 3.4.3 GRP78 expression

**FIGURE 7:**
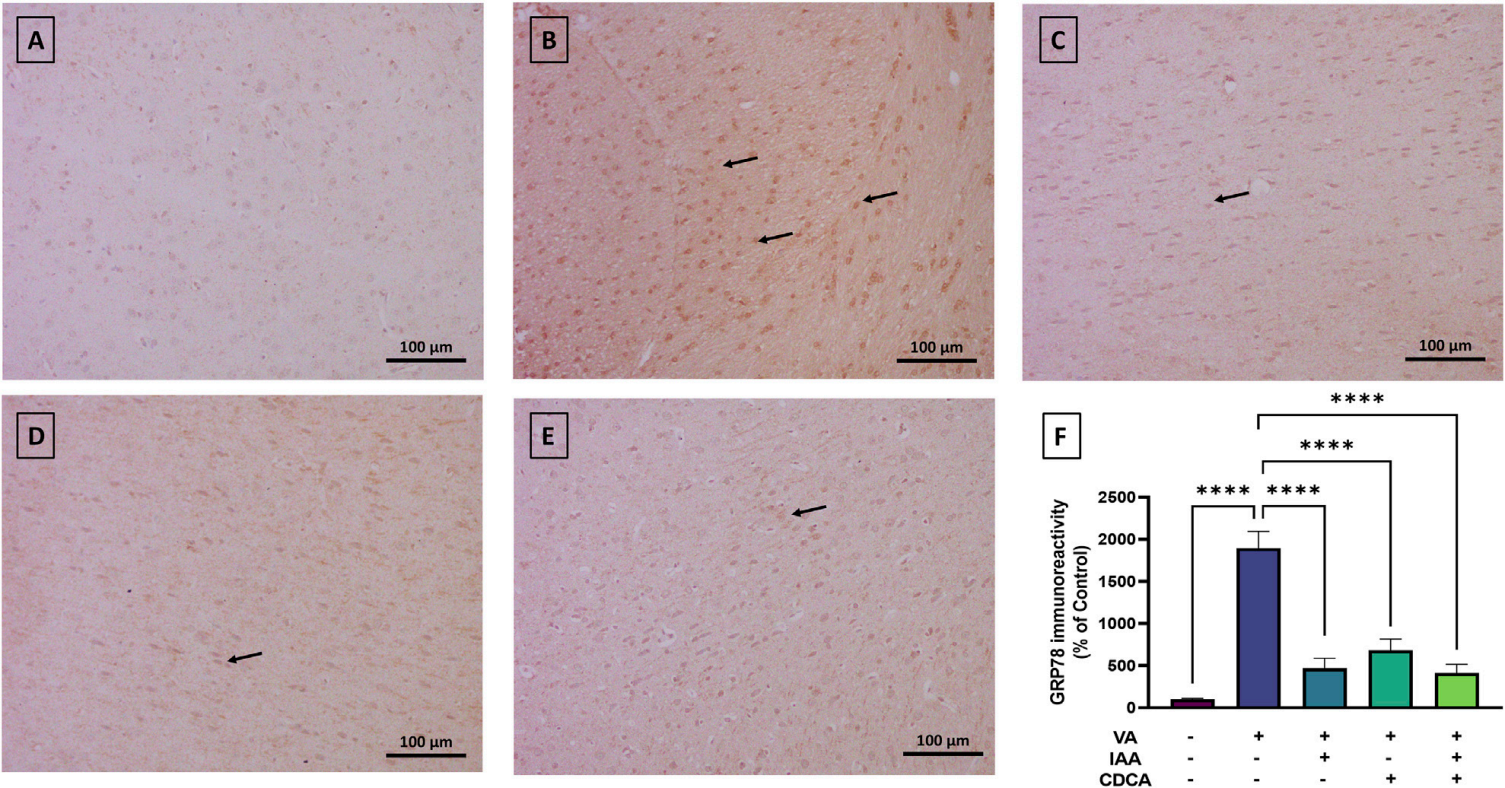
Immunoreactivity of GRP78 in brain cortex during VA neurotoxicity and after IAA and CDCA treatments. Scale bar: 100 μm, magnification ×200. **(A)** Brain cortex of normal control rats showing normal immune reactive signal for GRP78. **(B)** Brain cortex of rats intoxicated with VA exhibiting a strong upregulation in immunoreactivity (arrows). **(C–E)** Brain cortex of VA-intoxicated rats concurrently treated with **(C)** IAA, **(D)** CDCA, and **(E)** IAA + CDCA demonstrating significant reductions in VA-induced upregulation in GRP78 expression. **(F)** Quantification of GRP78 expression in different treatment groups using ImageJ. Values are expressed as mean ± SEM. ****P < 0.0001, (*n* = 4). GRP78: glucose-regulated protein 78, VA: valproic acid, IAA: indole-3-acetic acid, and CDCA: chenodeoxycholic acid.

To further elucidate the effect of VA on cortical neurons, markers of ER stress were assessed. GRP78 expression showed normal immunoreactivity in control rats, while VA exposure resulted in a significant increase in protein expression, as shown in Figure 7 (****P < 0.0001). All treatment groups reversed this increase with a dramatic reduction in protein expression (****P < 0.0001).

#### 3.4.4 ATF6 expression

**FIGURE 8:**
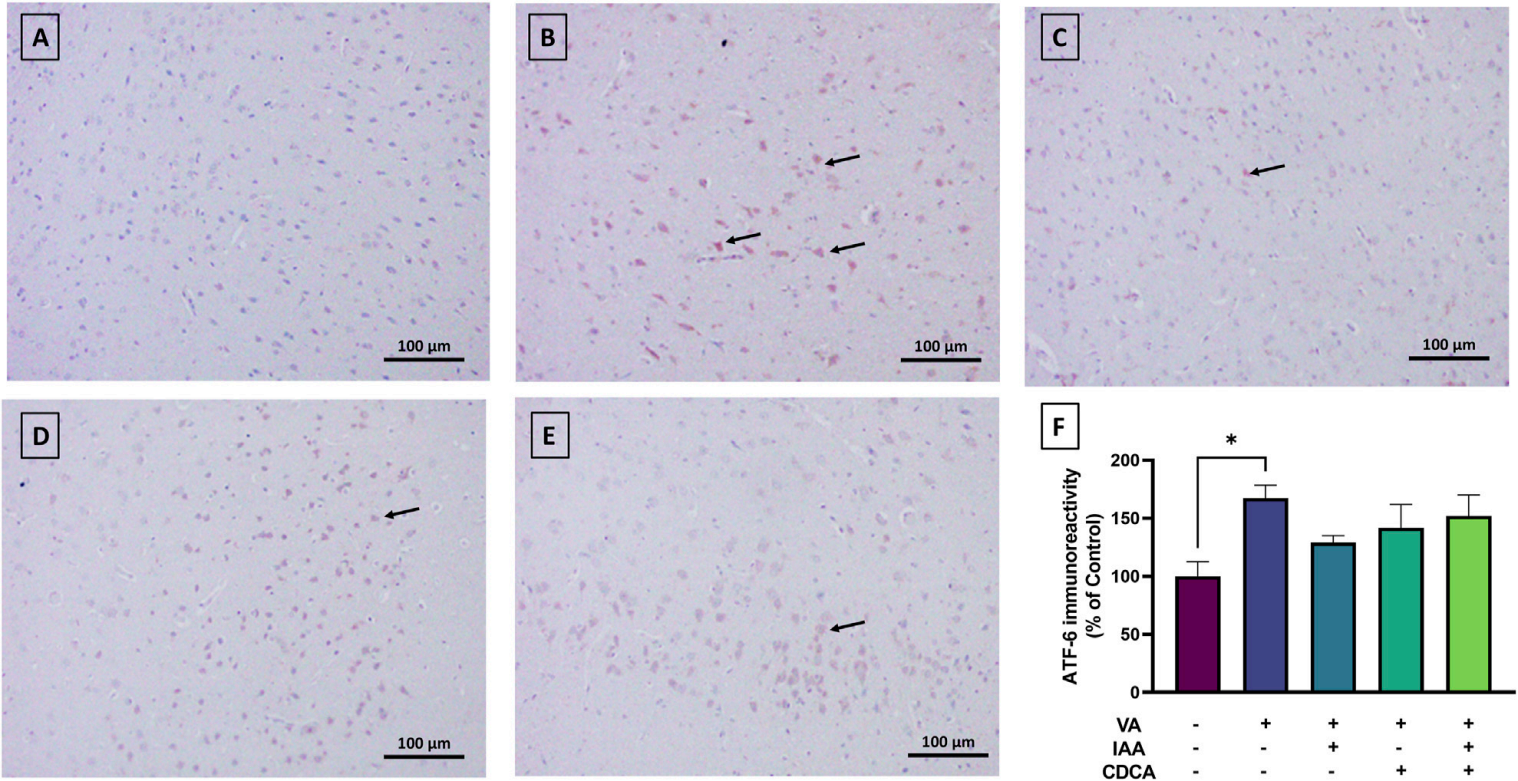
Immunoreactivity of ATF6 in brain cortex during VA neurotoxicity and after IAA and CDCA treatments. Scale bar: 100 μm, magnification ×200. **(A)** Brain cortex of normal control rats showing normal immune reactive signal for ATF6. **(B)** Brain cortex of rats intoxicated with VA exhibiting a mild upregulation in immunoreactivity (arrows). **(C–E)** Brain cortex of VA-intoxicated rats concurrently treated with **(C)** IAA. **(D)** CDCA, and **(E)** IAA + CDCA demonstrating some reductions in VA-induced upregulation in ATF6 expression. **(F)** Quantification of ATF6 expression in different treatment groups using ImageJ. Values are expressed as mean ± SEM. *P < 0.05, (*n* = 4). ATF-6: transcription factor 6, VA: valproic acid, IAA: indole-3-acetic acid, and CDCA: chenodeoxycholic acid.

Cortical brain sections from the normal control group revealed very low ATF6 expression (Figure 8). The use of VA slightly increased the immunoreactive signal relative to control rats (*P < 0.05). Treatment with IAA or CDCA in conjunction with VA reduced the immunoreactivity but the results were not significant (P > 0.05).

#### 3.4.5 CHOP expression

**FIGURE 9:**
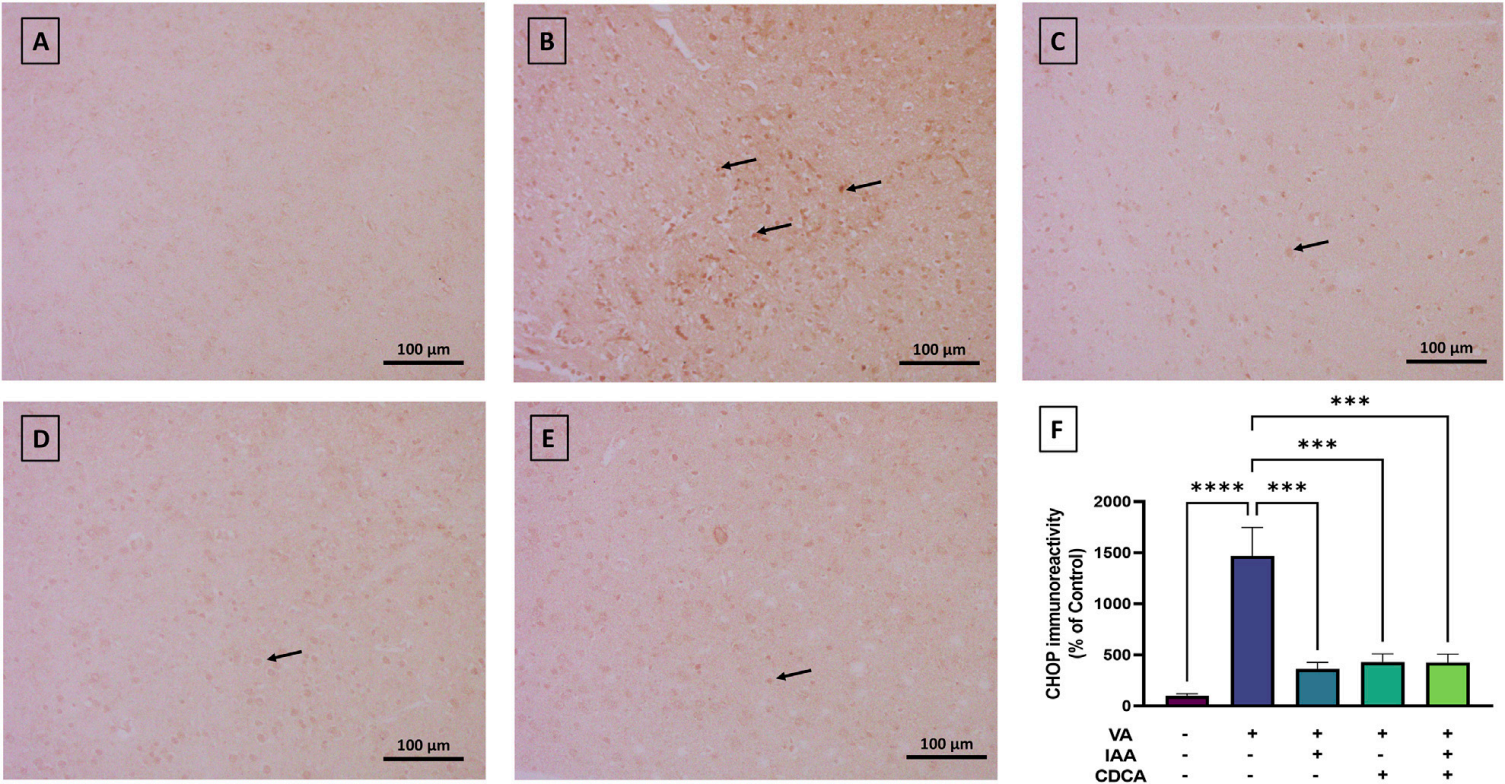
Immunoreactivity of CHOP in brain cortex during VA neurotoxicity and after IAA and CDCA treatments. Scale bar: 100 μm, magnification ×200. **(A)** Brain cortex of normal control rats showing normal immune reactive signal for CHOP. **(B)** Brain cortex of rats intoxicated with VA exhibiting a strong upregulation in immunoreactivity (arrows). **(C–E)** Brain cortex of VA-intoxicated rats concurrently treated with **(C)** IAA, **(D)** CDCA, and **(E)** IAA + CDCA demonstrating significant reductions in VA-induced upregulation in CHOP expression. **(F)** Quantification of CHOP expression in different treatment groups using ImageJ. Values are expressed as mean ± SEM. ****P < 0.0001, ***P < 0.001, (*n* = 4). CHOP: C/EBP homologous protein, VA: valproic acid, IAA: indole-3-acetic acid, and CDCA: chenodeoxycholic acid.

As shown in Figure 9, CHOP immunoreactivity was mild in control rats, while VA exposure showed a strong immunoreactive signal and resulted in a significant increase in protein expression (****P < 0.0001). All treatment groups reversed this increase with a dramatic reduction in CHOP expression (***P < 0.001).

## 4 Discussion

VA is a highly effective medication used to treat seizures, bipolar disorder, migraines, and anxiety (Patel and Nagalli, 2024; Turkyilmaz et al., 2021). However, despite its benefits, long-term use of VA, especially in high doses, above 60 mg/kg/day (Rahman et al., 2024), is linked to numerous adverse effects, including hepatotoxicity and neurological damage (Tunali et al., 2020). Given the significance of our research, further investigation into the mechanisms of VA-induced neuronal toxicity is crucial. Our study aims to address this need by examining these mechanisms and the associated downstream signaling pathways. In addition, it further explores the neuroprotective potentials of IAA and CDCA during such toxicity and their roles in oxidative stress, inflammation, and ER stress in cortical neurons. Yet, no prior research has examined the effects of IAA or CDCA on the neuronal histopathological, oxidative, and inflammatory changes associated with VA exposure, highlighting the novelty and importance of our work. This study explores the novel neuroprotective potential of IAA and CDCA against VA-induced neurotoxicity in rat cortical neurons, demonstrating for the first time that these compounds can mitigate VA-mediated oxidative stress, inflammation, and endoplasmic reticulum (ER) stress. VA has been reported to affect cortical and hippocampal neurons (Taleb et al., 2021; Chen et al., 2000). Cortical regions were selected for this study due to the larger size of the cortex, and importantly, to avoid the complexities of analyzing mixed brain regions. Brain regions have distinct protein compositions vital for their specialized functions. Combining these regions for analysis masks regional differences dilutes key protein signals, and prevents accurate assessment of drug responses on protein expression.

Chronic use of VA, especially with high doses, exerts toxic effects on cortical neurons through multiple mechanisms. VA-induced hyperammonemia, resulting from decreased carnitine levels, can lead to encephalopathy, and cerebral edema and these may alter VA metabolism allowing more toxic metabolites to penetrate the brain (Patel and Nagalli, 2024). Mitochondrial dysfunction is also involved by inhibiting fatty acid beta-oxidation and contributes to cellular injury (Patel and Nagalli, 2024). VA overdose also increases oxidative stress, inflammation, and neuronal damage. Similarly, our results revealed VA-induced cortical neuronal damage as evidenced by pyknosis and dark pigmentation of pyramidal neurons primarily in layer III of the rat cortex. The cortex is the main brain area involved in VA-induced neuronal damage (Chen et al., 2000). Direct toxic effects of VA or accumulation of its toxic metabolites in this brain region could be the main cause of such damage. In addition, VA causes neuronal cell death via a calpain-dependent necroptosis mechanism, which activates c-Jun N-terminal kinase 1 (JNK1) and receptor-interacting protein 1 (RIP-1), resulting in mitochondrial malfunction and DNA degradation (Bollino et al., 2015). Consistent with our findings, recent work showed similar histopathological alterations associated with VA neuronal toxicity showed increased neurons exhibited severe degeneration, accompanied by intense hyperemia in the blood vessels and substantial gliosis in the surrounding tissue (Terim Kapakin et al., 2024).

Oxidative stress arises from an imbalance between increased production of harmful free radicals, like reactive oxygen species (ROS), and insufficient antioxidant defenses. This imbalance can damage neurons, leading to consequences such as lipid peroxidation of neuronal membranes (Ogut et al., 2022; Jaganjac et al., 2022). Consequently, disrupted redox homeostasis can contribute to neuronal degeneration and loss, as observed in VA neurotoxicity. Specifically, ROS can attack neuronal membranes, impair protein function, and cause DNA mutations. This damage also extends to blood vessels, promoting inflammation and further tissue injury (Ogut et al., 2022).

In parallel with the histopathological changes, chronic VA exposure in rats showed reduced levels of the antioxidants GSH and SOD in the brain cortex, indicative of oxidative stress. GSH is a non-enzymatic antioxidant that can directly neutralize various reactive species while SOD is an enzymatic antioxidant that specifically targets superoxide radicals, converting them into less harmful molecules (Aoyama, 2021; Lee et al., 2020). Neurons are especially vulnerable to oxidative stress due to their limited repair capacity and high metabolic rate, which results in increased ROS production. VA induced oxidative stress in the brain by increasing malondialdehyde (MDA) and reducing antioxidants catalase, SOD, and glutathione peroxidase (GPx) (Kaewngam et al., 2024; Adewole et al., 2023). The use of 200 and 400 mg/kg of VA in rats caused a significant increase in MDA with a decrease in GSH in the brain (Hussein et al., 2021). Furthermore, VA downregulated the expression of nuclear factor erythroid 2-related factor 2 (Nrf2), a transcription factor crucial for oxidative stress defense and maintenance of redox homeostasis (Kaewngam et al., 2024; Hussein et al., 2021; Jin et al., 2014).

On the other hand, the use of IAA, CDCA, or their combined treatments protected the neurons, minimized neuronal loss, and reversed histopathological alterations and oxidative stress that occurred after VA exposure. Interestingly, IAA proved more effective than CDCA or the combined treatment against the morphological and biochemical changes that occur after VA exposure. These results aligned with recent studies that reported the antioxidant effects of IAA on other organs as it reduced MDA and increased SOD, GSH, and heme oxygenase-1 (HO-1) and was effective against VA-induced liver and renal toxicities (Aljarboa et al., 2023; Alhusaini et al., 2024). It is also an effective antioxidant against high-fat diet-inducing non-alcoholic fatty liver disease (NAFLD) (Ji et al., 2019). Moreover, Ji et al. showed that IAA exhibits antioxidant and free radical scavenging activity and induces HO-1 production in lipopolysaccharides (LPS)-stimulated RAW264.7 macrophages, which corroborates our results (Ji et al., 2020). CDCA has also demonstrated antioxidant activity, particularly against VA-induced liver and renal toxicities (Aljarboa et al., 2023; Alhusaini et al., 2024) and aluminum chloride-induced neurotoxicity (Bingül et al., 2024). Based on these findings, IAA and CDCA, when administered individually, may have antioxidant properties that could potentially reverse VA-induced neurotoxicity.

Given the aforementioned connection between oxidative stress and VA-induced neurotoxicity, neuroinflammation is a subsequent pathological response. Our results demonstrated VA-induced neuronal inflammation in the cortical tissue, as evidenced by elevated levels of the inflammatory cytokines; TNF-α and IL-6 and upregulation of TLR4/NF-κB signaling. These findings correspond with prior research showing that VA induced the proinflammatory cytokines (Adewole et al., 2023), activated NF-κB (Jin et al., 2014), and upregulated TLR4 (Aljarboa et al., 2023). Moreover, VA exposure increased hepatic expression of TLR4 which can be attributed to oxidative stress and tissue inflammation (Aljarboa et al., 2023). Consistent with the established link between VA and neuronal TLR4/NF-κB signaling, recent work by Farrag et al. demonstrated that VA-exposed rats exhibited autism-like behaviors, alongside elevated levels of TLR4, NF-κB, and ROS, as well as induced neuronal apoptosis (Farrag et al., 2024).

To some extent, the use of VA with IAA and/or CDCA ameliorated VA-induced inflammation and TLR4/NF-κB signaling activation. This amelioration primarily observed IAA more than CDCA and combined treatments. IAA has anti-inflammatory and antioxidant properties, it possesses protective effects against long-term inflammation in the ankylosing spondylitis (AS) mouse model by inhibiting pro-inflammatory cytokines; TNF-α, IL-6, IL-17A, and IL-23 while promoting anti-inflammatory IL-10 (Shen et al., 2022). In mice fed a high-fat diet, IAA treatment resulted in attenuated macrophage infiltration and the expression of monocyte chemoattractant protein-1 (MCP-1) and TNF-α, suggesting an attenuation of liver inflammation (Ji et al., 2019). IAA also counteracted LPS induced inflammatory response in RAW264.7 macrophages by downregulating IL-1β, IL-6, MCP-1, and NF-κB expression (Ji et al., 2020). Additionally, CDCA has demonstrated anti-inflammatory activity by reducing inflammatory cytokines levels, particularly against VA-induced liver and renal toxicities (Aljarboa et al., 2023; Alhusaini et al., 2024). The activation of FXR by CDCA in chronic periarteritis reduces proinflammatory factors (Cao et al., 2021). Furthermore, CDCA can impact the composition of gut microbiota. This modulation may contribute to its neuroprotective effects, as a balanced gut microbiota is crucial for maintaining overall health and can influence the neuroinflammatory process (Tian et al., 2023).

Besides inflammation and TLR4/NF-κB signaling, ER stress markers were assessed during VA toxicity and after treatments. ER stress is a key mechanism that is implicated in neurological diseases, particularly in neurodegenerative diseases including Alzheimer’s disease and Parkinson’s disease (Ajoolabady et al., 2022; Hasan et al., 2024), during heavy metal toxicity (Pyatha et al., 2022), and after neurotoxin exposure (Selvaraj et al., 2012). The ER is the cellular compartment where proteins undergo folding and modification. When the cell is exposed to stress, the ER can become overwhelmed by an accumulation of improperly folded proteins. This triggers the unfolded protein response (UPR), a cellular defense mechanism, that aims to restore protein homeostasis by activating key ER stress sensors; protein kinase R-like ER kinase (PERK), activating transcription factor 6 (ATF6), and inositol-requiring enzyme 1 (IRE1) (Hasan et al., 2024; Roussel et al., 2013; Shi et al., 2021). Under normal cell homeostasis, these sensors remain inactive due to their binding to the chaperone protein GRP78. During UPR, however, these sensors become active as misfolded proteins increase and bind to GRP78, disrupting its ability to facilitate proper protein folding. ATF6 is a transcription factor activated during ER stress, which upregulates CHOP (a pro-apoptotic gene) and other ER stress-associated genes, including GRP78, GRP94, and X-box binding protein 1 (XBP1) (Hu et al., 2018; Lei et al., 2024).

Previous studies have shown that regular doses of VA attenuated ER stress and protected neurons (Li et al., 2017; Fu et al., 2019). Short-term administration of VA alleviated ER stress and following traumatic brain injury by inhibition of the GRP78-ATF6-CHOP signaling pathway (Chen et al., 2024). Nevertheless, our research demonstrated that chronic exposure to VA increased the expression of GRP78, ATF-6, and CHOP, which are indicators of ER stress. Consistent with our findings, studies have shown that chronic exposure to VA in rats upregulated gene and protein expression of GRP78, the main regulator for ER stress, primarily in the cortical brain region (Chen et al., 2000; Wang et al., 1999). Besides that, exposure to VA increased the expression of other ER stress proteins such as GRP94 and calreticulin (Chen et al., 2000). These observations, however, underscore the complex relationship between VA and ER stress, revealing that the effects of VA are critically dependent on both exposure duration and the specific cell type, including its origin and diversity.

Alternatively, the use of IAA and/or CDCA showed similar effectiveness against VP-associated ER stress by reducing GRP78 and CHOP expressions. The limited effect of these drugs on ATF6 expression may be due to its low basal expression levels or the involvement of other ER stress sensors, such as PERK and IRE1. Despite the absence of direct investigations into the effects of IAA and CDCA on neuronal ER stress, several studies have documented their complex influence on ER stress responses in other experimental models. For example, IAA confers protection against endoplasmic ER stress in *Caenorhabditis elegans*. This protection of IAA is primarily mediated through the IRE-1/XBP-1 branch of the UPR (Bhoi et al., 2021). CDCA, on the other hand, can suppress ER stress induced by soybean oil *in vivo* through its agonistic activity on FXR. This suppression is evidenced by significant reductions in ER stress markers such as GRP78, CHOP, XBP1S, ATF4, PERK, and ATF6 (Du et al., 2022). However, CDCA does not always alleviate ER stress, it can paradoxically induce ER stress markers and cell death, and possibly be influenced by intracellular calcium levels and ROS generation (Adachi et al., 2014). Thus, the ability of CDCA to modulate ER stress is dependent on the specific cellular type and experimental conditions.

Our findings indicate that combined treatment with IAA and CDCA offered less protection against VA-induced neurotoxicity compared to IAA alone, particularly when assessing brain histology and oxidative stress markers. This result was unexpected, as both compounds have demonstrated protective effects in other tissues and models of drug toxicity or tissue injury (Ji et al., 2019; Kim et al., 2017; Chen et al., 2022; Hu et al., 2012). Similarly, the combination failed to show additive or synergistic benefits in some measures of VA-induced liver toxicity (Aljarboa et al., 2023). One possible explanation is a pharmacokinetic interaction between the two compounds. CDCA, a bile acid, could influence IAA absorption in the gastrointestinal tract, potentially altering its bioavailability (Torrent Rodríguez et al., 2024) especially considering the oral gavage administration route. Further mechanistic studies are necessary to fully elucidate the complex interplay between IAA and CDCA.

Although our preclinical study of IAA and CDCA in a model of VA-induced brain injury yielded promising results, translating results to humans requires further investigation. Currently, there are no published clinical studies evaluating the therapeutic use of IAA in humans. CDCA, on the other hand, has shown some potential for a rare metabolic disorder; cerebrotendinous xanthomatosis (CTX) (DeBarber et al., 2024). Therefore, the clinical relevance of our findings is limited at this stage. A thorough understanding of the pharmacokinetic, pharmacodynamic, and safety profiles of both IAA and CDCA is essential before considering their potential clinical applications.

This study provides initial evidence that IAA and/or CDCA protect against VA-induced neuronal toxicity by downregulating GRP78, ATF6, and CHOP, key proteins in the ER stress pathway. This effect is corroborated by the downregulation of the TLR4/NF-κB signaling pathway. While our findings strongly suggest a protective role for IAA and CDCA against VA-induced neuronal ER stress, this work has some limitations, including the small sample size and focus on the cortical brain region. Future studies using larger sample sizes and examining other brain areas are necessary to confirm these findings and further elucidate the precise mechanisms and direct molecular interactions involved, particularly those involving CHOP, ATF6, and GRP78.

## 5 Conclusion

To sum up, these results introduce compelling evidence for the deleterious effect of VA-induced neurotoxicity on brain tissue and the protective effects of IAA and CDCA. While VA is capable of inducing oxidative stress, inflammation, and ER stress under our conditions of high dosage and chronic exposure, it may also confer neuroprotective benefits in other contexts of neuronal damage, thus illustrating the complexity of its pharmacological actions. IAA and CDCA treatment mitigated VA-induced neuronal oxidative stress, inflammation, and ER stress. This was demonstrated by improved oxidative stress markers, reduced inflammatory cytokine levels, and downregulation of TLR4, NF-κB, and ER stress markers, suggesting a potential therapeutic role for IAA and CDCA in VA-induced neurotoxicity. Promising preclinical results with IAA and CDCA in a VA-induced brain injury model require further investigation before human translation. Additional research is warranted to elucidate further the underlying neuroprotective mechanisms of IAA and CDCA on alternative ER sensors, PERK and IRE1, and investigate their potential in other neurotoxicity models.

## Data Availability

The original contributions presented in the study are included in the article/Supplementary Material, further inquiries can be directed to the corresponding author.

## Glossary

ABC: Avidin-Biotin Complex
AS: Ankylosing spondylitis
ATF-6: Activating transcription factor 6
CAT: Catalase
CDCA: Chenodeoxycholic acid
CHOP: C/EBP homologous protein
CMC: Carboxymethylcellulose
CNS: Central nervous system
DAB: Diaminobenzidine
ELISA: Enzyme-linked immunosorbent assay
ER: Endoplasmic reticulum
FXR: Farnesoid X receptor
GABA: γ-aminobutyric acid
GPx: Glutathione peroxidase
GRP78: Glucose-regulated protein 78
GSH: Glutathione
H&E: Hematoxylin and eosin
HO-1: Heme oxygenase-1
IAA: Indole-3-acetic acid
IHC: Immunohistochemical
IL-6: Interleukin-6
IRE1: Inositol-requiring enzyme 1
KSU: King Saud University
LPO: Lipid peroxidation
LPS: Lipopolysaccharides
MCP-1: Monocyte chemoattractant protein-1
MDA: Malondialdehyde
NAFLD: Non-alcoholic fatty liver disease
NF-κB: Nuclear factor kappa B
NP-SH: n-protein thiol activity
Nrf2: Nuclear factor erythroid-related factor 2
PBS: Phosphate-buffered saline
PERK: Protein kinase R-like ER kinase
SEM: Standard error of the mean
SOD: Superoxide dismutase
TLR4: Toll-like receptor 4
TNFα: Tumor necrosis factor-alpha
UPR: Unfolded protein response
VA: Valproic acid
XBP1: X-box binding protein 1
